# Mucormycosis mimicking portal hypertensive haemorrhage as a complication of alcoholic liver cirrhosis: a case report

**DOI:** 10.1186/s12879-023-08220-0

**Published:** 2024-01-29

**Authors:** Ran Ji

**Affiliations:** Department of the Surgical Intensive Care Unit (SICU), The Second Affiliated Hospital Zhejiang, University School of Medicine, NO. 88 Jiefang Road,Shangcheng District Hangzhou, 310009 Zhejiang, China

**Keywords:** Mucormycosis, Upper gastrointestinal bleeding, Alcoholic liver cirrhosis, Immunosuppression

## Abstract

Mucor is a rare cause of gastrointestinal ulcers. This case describes a case of mucormycosis that occurred in a patient with liver cirrhosis who was hospitalized to accept a splenectomy for traumatic splenic rupture. During the perioperative period, the patient developed upper gastrointestinal bleeding(UGIB), which was diagnosed as mucormycosis-related gastric ulcer according to gastroscopy. Patients with liver cirrhosis often get UGIB for Portal hypertension, but they also can develop UGIB for multiple other reasons, including infectious ulcers for immunosuppression. The case emphasizes the importance of excluding fungal-induced ulcer haemorrhage before diagnosing Portal hypertensive-induced variceal haemorrhage in patients with liver cirrhosis.

## Introduction

Mucormycosis is a rare invasive fungal disease caused by a few filamentous moulds within the order of [1]. It needs urgent intervention because of the nature of progress and [2]. According to a prospective multicenter study in India, immunocompromised patients are more prone to acquire mucormycosis and the mortality of which is about 46.7%. Based on the site of infection, the incidence of gastrointestinal mucormycosis is about 6.4% [3]. According to the involved organs, mucormycosis is classified into six types. It includes gastrointestinal (GI), pulmonary, cutaneous, rhino-orbital-cerebral mucormycosis (ROCM), disseminated, and mucormycosis of uncommon [4]. Compared with the other types, primary gastrointestinal mucormycosis is rare and is encountered primarily in immunocompromised [5]. The mortality rates range from 40–85% [6]. The most common site for GI mucormycosis is the stomach, accounting for 57.5% of cases, followed by the colon at 32.2%, and the small intestine at 10.3% [7, 8]. Patients with liver cirrhosis are usually weak in immune [9] which are impaired to defend against infection. It often correlates with liver injury, bacterial translocation and organ failure [10]. Some studies reported cases of mucormycosis in patients with liver cirrhosis [11–13]. Upper gastrointestinal haemorrhage is the most common complication of liver cirrhosis, often seen in intensive units (ICU), which is usually caused by portal hypertension, and affecting clinical [14–16]. However, UGIB also is induced by a gastric ulcer as a complication of liver [17, 18]. As a result, when patients with liver cirrhosis occur haematemesis accompanied by a haemoglobin decrease, the cause is easily misdiagnosed in patients with liver cirrhosis because of stereotypes. It is essential to differentiate the causes that induce upper gastrointestinal haemorrhage in patients with liver cirrhosis.

This study presents a case report of mucormycosis-induced gastric ulcer with haemorrhage. This case occurs in a patient with alcoholic liver cirrhosis who receives a splenectomy after a traumatic splenic rupture.

## Case report

**Fig. 1:**
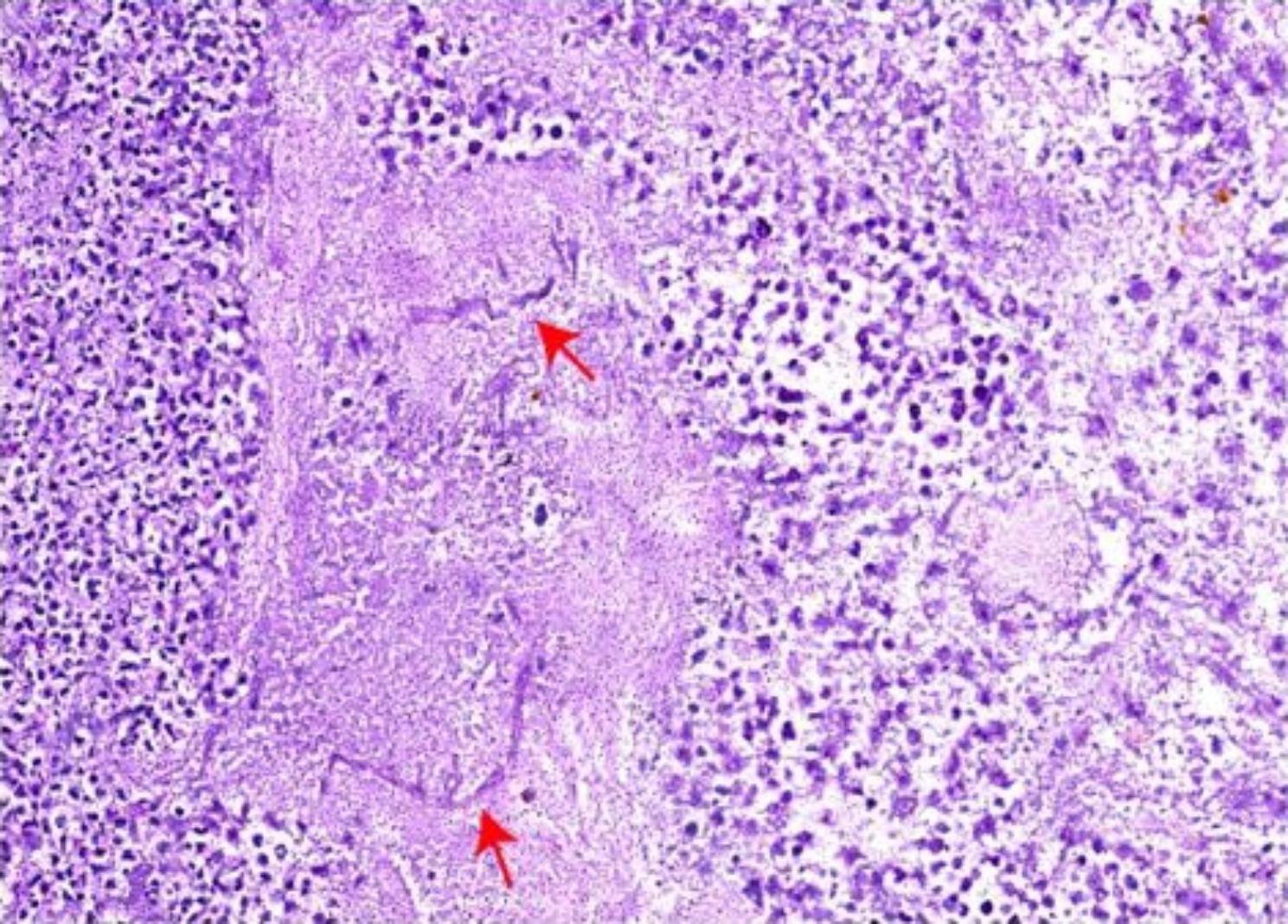
(5–20 μm in diameter) thin-walled, ribbon-like hyphae with few septations and right-angle branching suggestive of mucormycosis were demonstrated by PAS stain

**Fig. 2:**
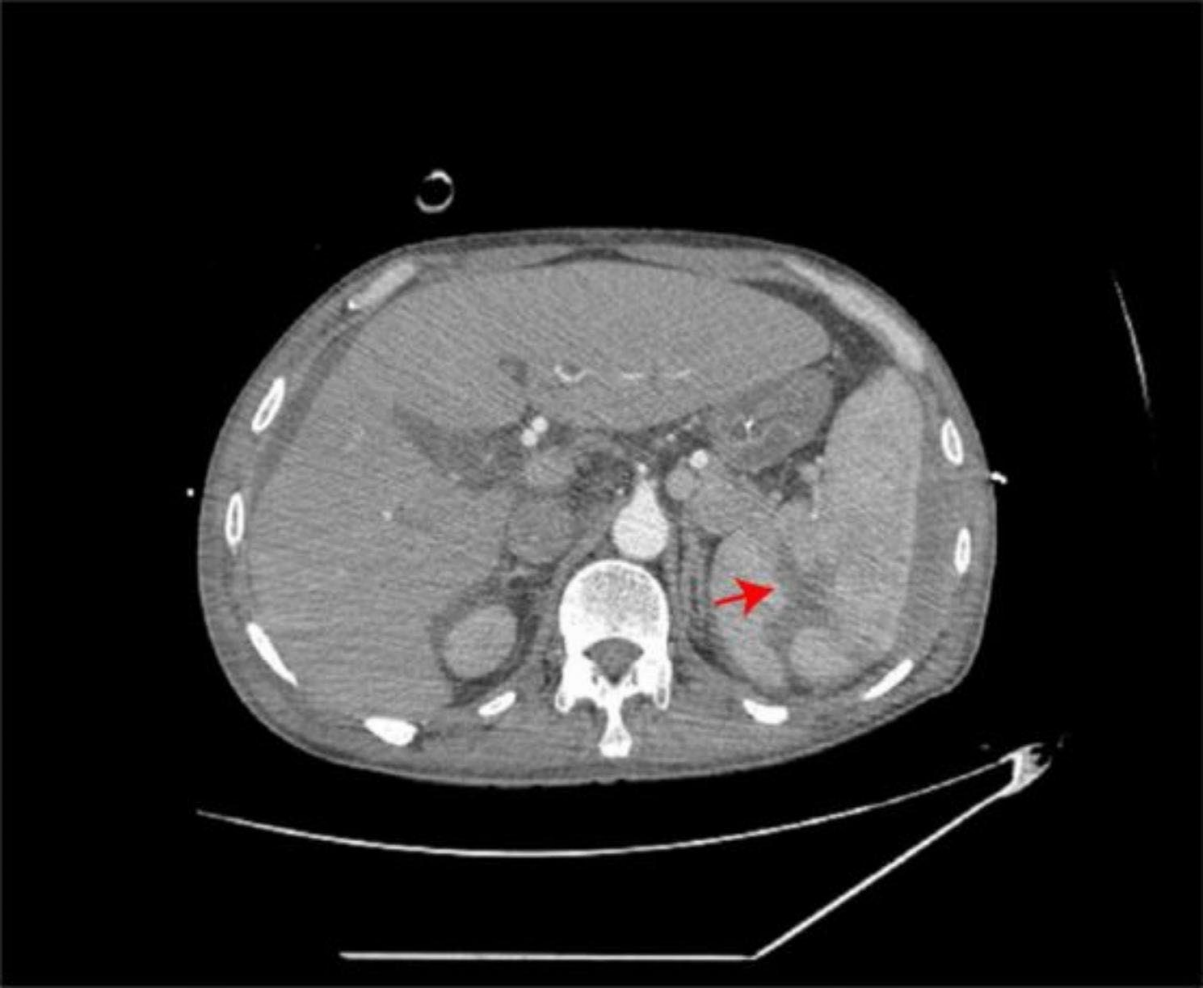
The abdomen-enhanced CT scan showed: a spleen laceration

**Fig. 3:**
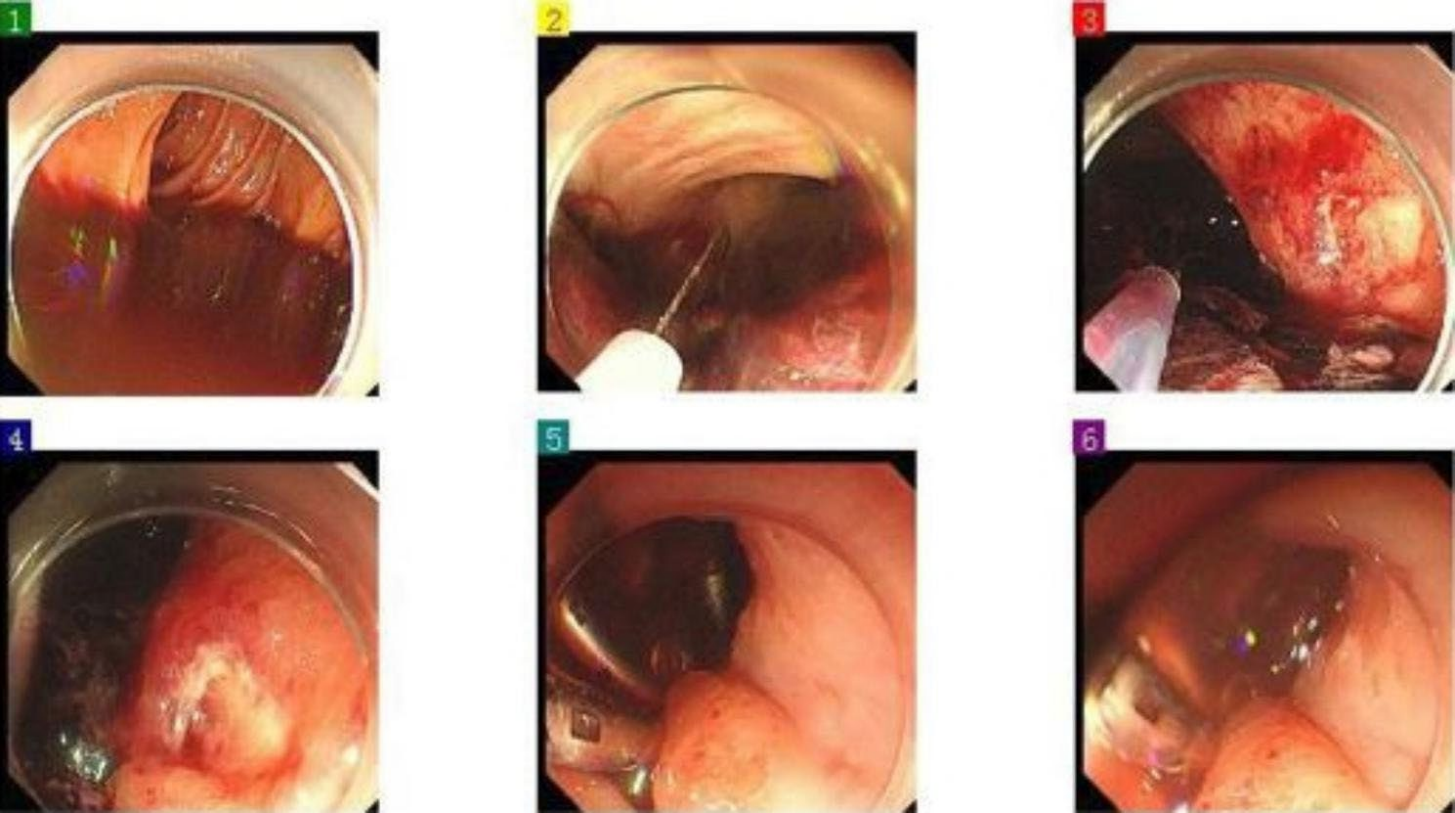
Gastroscopy showed: Blood can be seen in the oesophagal cavity, and a large amount of old bleeding and blood clots can be seen in the cardia, gastric cavity, and duodenum. Ulcerative changes can be seen near the cardia on the upper posterior wall of the gastric body, with a small amount of bleeding

On 7/29/2022, a 57-year-old man underwent surgery for a splenectomy due to a spleen rupture following nine days of conservative treatment. Nine days before, the patient had fallen from a motorbike and received a diagnosis of traumatic splenic rupture through CT scanning. The patient’s medical history revealed a previous spleen rupture occurrence nine years ago, which had resolved with conservative treatments. The patient’s medical history also indicated that he had developed Child-Pugh A liver cirrhosis some years ago. He did not have any gastroscopies or receive any treatment for it. On arrival at our centre, his vital signs showed blood pressure of 138/77 mmHg, heart rate of 91 bpm, oxygen saturation of 100% on ventilation volume of 450ml, a body temperature of 37.6℃, and respiratory rate of 18 breaths per minute. His initial Glasgow coma scale was E4VTM6 and initial physical examinations revealed remarkable left upper abdominal tenderness, rebound tenderness and mild muscle tension. The abdomen-enhanced CT scan showed a spleen laceration(Fig. 1). The Blood test showed Haemoglobin (Hb) ( 68 g/L), Platelet (Plt) (269 × 10^9/L), Procalcitonin (PCT) (1.53ng/mL) and C-Reactive Protein CRP(54.8 mg/L) were also increased. The Patient’s liver function test was irregular that ALT (32U/L), AST( 83U/L), TBIL(99.3µmol/L), DBIL(36.5µmol/L) and IDBIL (24.1µmol/L). The Patient’s coagulation was also abnormal that APTT (44.8s)、PT (17.5s)、D-dimer (7050 µg/L FEU), and INR (1.40). As cholecystitis and spleen laceration was suspected, Imipenem/cilastatin was initiated, followed by fluid resuscitation, liver protection (including Ademetionine 1,4-Butanedisulfonate, Glutathione and L-Ornithine-L-Aspartate), Proton-pump inhibitor (PPI), and other supportive treatments. After supportive treatments in the ICU for 5 days, he received surgery of Splenectomy, Cholecystectomy and Choledocholithotomy. After surgery, he continue to be monitored in the ICU. On hospital day 11th, he appeared bloody stools and hematemesis accompanied by hypovolemic shock (Hb 34 g/L; NE 0.05 ug/kg min). Acute upper gastrointestinal bleeding was suspected, and the patient received an emergency gastroscopy immediately (Fig. 2). Under the gastroscopy, he got an ulcer at the gastric cardia covered by a lot of old blood clot, which was clamped by Titanium clips at last. After that, he got a plasma and red blood transfusion to improve coagulation and haemoglobin. But the patient received an emergency gastrectomy for UGIB on the fifth day after the gastroscopy. After the operation, the patient received continued anti-infective treatment (Piperacillin Sodium and Tazobactam Sodium, 4.5 g, every 8 h), fluid rehydration, nutritional support, and sputum reduction. After a few days, the pathology report showed that a gastric ulcer was produced by mucormycosis (Fig. 3). According to the report, Posaconazole was added to fight mucor. The patient received Posaconazole (100 mg 2 times per day) for instillation in a feeding tube. Despite initial improvements, the patient developed liver injury 【TBIL (110.4µmol/L), DBIL(64.2µmol/L), IDBIL (46.2µmol/L) 】, sepsis (SOFA score 19) and acute respiratory distress failure (P/F = 43.2 mmHg). The patient’s family members abandoned any treatment for him and soon left the hospital. His death was confirmed a few days after follow-up.

## Discussion

It is believed that this is a rare case report of mucormycosis-related UGIB that occurred in a patient with alcoholic liver cirrhosis during the perioperative splenectomy period. It can be concluded that this case occurred GI mucormycosis for Four reasons. First, Mucormycosis is an opportunistic fungal infection that is encountered in immune-compromised [19]. Alcoholic liver cirrhosis is closed related to immune function [20] by activating some immune cells. Seung Hyoung Lee and so on reported a similar case in 2014 [21]. Second, gastrointestinal dysfunction is essential for microbial colonization. Gastrointestinal dysfunction is a common complication of liver cirrhosis because of dysautonomia, hormone alteration and portal [22–24]. Third, PPI is widely used to increase PH in the stomach. It can also promote Mucor colonization. Forth, the integrity of the mucosal barrier also plays a vital role in GI mucormycosis. The mucosal barrier is often weakened in liver cirrhosis patients. Besides the secretion of bile and gastric juice decrease, liver cirrhosis-related portal hypertension can decrease the expression of occludin and claudin-1, both of which participate in the integrity of the mucosal [25]. In ICU, Gastrointestinal feeding tube placement can also increase the risk of barrier damage.

Hypertension-induced variceal haemorrhage is believed to be one of the most common complications of liver cirrhosis, the onset was severe in its clinical course. Also, peptic ulcers, portal hypertensive gastropathy (PHG), and some other reasons can cause upper gastrointestinal haemorrhage, as varices are. According to research, about 40% of patients developed UGIB for reasons not related to portal hypertension in cirrhotic patients with [26]. Due to similar clinical symptoms of haematemesis to portal hypertension, it is difficult to diagnose the alcoholic liver cirrhosis-induced UGIB at the bedside [27]. The patient doesn’t present obvious hypertension-related clinical signs upon admission to ICU, like splenomegaly, ascites, etc. But the emergency endoscopy results supported peptic ulcer bleeding, and there were no apparent gastric oesophagal varices discovered. Therefore, it was more natural that his UGIB was complicated by a gastric ulcer, not by portal hypertension.

In conclusion, mucormycosis-related upper gastrointestinal bleeding mimics hypertension-induced variceal haemorrhage. It suggested that mucormycosis might be a different diagnosis of UGIB among patients with liver cirrhosis. Mucormycosis-related ulcers can be diagnosed by gastroscopy and can be treated with antifungal agents and operative treatment.

## Data Availability

The datasets used and/or analysed during the current study are available from the corresponding author upon reasonable request.
